# Rapid Flow Cytometry Detection of a Single Viable *Escherichia coli* O157:H7 Cell in Raw Spinach Using a Simplified Sample Preparation Technique

**DOI:** 10.3389/fmicb.2017.01493

**Published:** 2017-08-14

**Authors:** Anna J. Williams, Willie M. Cooper, Shawn Ramsaroop, Pierre Alusta, Dan A. Buzatu, Jon G. Wilkes

**Affiliations:** ^1^Division of Systems Biology, National Center for Toxicological Research, U.S. Food and Drug Administration, Jefferson AR, United States; ^2^Vivione Biosciences, LLC, Pine Bluff AR, United States

**Keywords:** sample preparation, food pathogens, bacterial detection in food, bacterial quantification, public health

## Abstract

Very low cell count detection of *Escherichia coli* O157:H7 in foods is critical, since an infective dose for this pathogen may be only 10 cells, and fewer still for vulnerable populations. A flow cytometer is able to detect and count individual cells of a target bacterium, in this case *E. coli* O157:H7. The challenge is to find the single cell in a complex matrix like raw spinach. To find that cell requires growing it as quickly as possible to a number sufficiently in excess of matrix background that identification is certain. The experimental design for this work was that of a U.S. Food and Drug Administration (FDA) In-House Level 3 validation executed in the technology’s originating laboratory. Using non-selective enrichment broth, 6.5 h incubation at 42°C, centrifugation for target cell concentration, and a highly selective *E. coli* O157 fluorescent antibody tag, the cytometry method proved more sensitive than a reference regulatory method (*p* = 0.01) for detecting a single target cell, one *E. coli* O157:H7 cell, in 25 g of spinach. It counted that cell’s daughters with at least 38× signal-to-noise ratio, analyzing 25 samples in total-time-to-results of 9 h.

## Introduction

Many foodborne outbreaks caused by *Escherichia coli* O157:H7 are associated with vegetables and fruits as a result of fecal contamination from domestic or wild animals at some phase during cultivation or handling (World Health Organization, 2016). Transmission to humans can occur through contacting or consuming contaminated raw foods, milk, or water (World Health Organization, 2016). To address this problem, regulators, food producers, retailers, and distributors need effective microbiological testing for quality control purposes (American Type Culture Collection [ATCC], 2012). Major requirements are for sensitive, specific, and rapid results. Most *E. coli* strains are harmless to the host (Souza et al., 1999), but those that produce Shiga-like toxins cause diarrheal and other significant diseases in humans (Paton et al., 1996). *E. coli* O157:H7, almost all strains of which produce Shiga-like toxins, is the serotype most often associated with pathogenicity and is implicated in many cases of foodborne illness in the United States (Doyle et al., 2006; Gehring et al., 2006). This organism can cause as high as 50% mortality in the elderly (The Center for Food Security and Public Health, 2009) and kidney failure in children (Reilly, 1998). It is estimated that ingesting only 10–100 cells of *E. coli* O157:H7 can cause foodborne illness (*Escherichia coli* O157:H7, 2017), but many people eat more than 25 g at a sitting and individuals with immature or compromised immune systems may be more sensitive to low level contamination. Thus detection of very low level contaminants is important.

A variety of rapid methods for detecting it in food have been developed to augment or replace plate count techniques (López-Campos et al., 2012). These include the enzyme-linked immunosorbent assay, pulsed-field gel electrophoresis, PCR, microarrays, and flow cytometry (López-Campos et al., 2012).

Objectives for rapid food pathogen detection include decreasing the time to results (TTR) and increasing surveillance throughput. Traditional methods typically take several days to detect and confirm the presence of a pathogen or toxin in a particular food (López-Campos et al., 2012). Since a constraint in food analysis involves existence of indigenous microorganisms, that are not necessarily harmful, however, their being there often hinders the selective identification and isolation of certain pathogens, which are usually present in low numbers (Mandal et al., 2011), it is important for the detection method to have the ability to remove the microorganisms from the food to the detection system (Hardin, 2011). Various strategies, using antibody-based as well as chemical and physical methods, have been developed to isolate pathogens from a variety of food sample matrices (Stevens and Jaykus, 2004; Bhunia, 2008). In the case of flow cytometry detection, isolation from the food is not necessary if physical occlusion of the flow channel can be eliminated and optical interference can be reduced. Occlusion in this work is eliminated by using a flow cell with a wide channel and by filtering sample suspension. Our previous publications have reported a number of sample preparation techniques that reduce optical interference (Wilkes et al., 2012; Buzatu et al., 2013, 2014; Williams et al., 2015).

Here we propose flow cytometry as an alternative instrumental platform. Its use produces results the same day as sample arrival, in the presence of the food matrix, and without the necessity of plate-based strain isolation before determination. That is, *E. coli* O157 can be detected and distinguished from non-pathogenic *E. coli* in perishable foods with fewer days TTR than other technologies allow.

The U.S. Food and Drug Administration (FDA) Bacteriological Analytical Manual (BAM) chapter for detection of Diarrheagenic *E. coli* O157:H7 in food, BAM 4a, specifies the use of PCR, although earlier plate-based methods based on selective media and assessment of colony morphology are also allowed when PCR instruments are not available (Feng et al., 2011). The BAM is the current regulatory or reference method used to detect *E. coli* O157:H7 in foods. The rationale for developing new microbiological methods is to detect the offending pathogen more quickly, with greater specificity, and with greater sensitivity.

The FDA has established protocols for new and rapid validation methods—FDA Methods Validation Guidelines for Microbial Pathogens (FDA Foods Program, Science, Research Steering Committee, 2011, p. 8). These exist in several levels, corresponding to the number of samples run and the number of laboratories involved in the validation exercise. For example, in a Level 1 validation, the originating laboratory characterizes the method with respect to linearity, specificity, inclusivity, and exclusivity. In a Level 2 validation, 20 samples are required to be analyzed by the originating laboratory: 10 non-inoculated and 10 inoculated with the target pathogen and refrigerated overnight (aged) to stress the cells. In a Level 3 validation, 25 samples must be analyzed by the originating laboratory and at least one collaborating laboratory. Refrigeration after inoculation before recovery, enrichment, and analysis must last 48–72 h. Preparatory to a multi-lab validation the agency describes an in-house variant executed by the originating laboratory. This is to fully prove all aspects of the process before potentially wasting time and money on a multi-lab test.

In this study, the rapid and regulatory methods were being analyzed based on parallel samples rather than samples split after enrichment because the two used different enrichment media, incubation temperatures, and non-selective enrichment periods. The different media and enrichment temperatures resulted from extensive optimization of the flow cytometry method intended to reduce TTR as much as possible, to obtain results within the same day rather than after overnight enrichment. Consequently, comparison of sensitivity was only possible based on recoveries—that is, by comparing the percentage of positive recoveries by the two methods for the nominally positive samples. The required sensitivity for the rapid method is equal or better recovery than an accepted regulatory method.

Comparison was made to a reference method (BAM Chapter 4a) for detection of *E. coli* O157:H7 in spinach. These experiments used a non-PCR reference method (Feng et al., 2011) because the laboratory originating the flow cytometry method did not have the equipment or expertise to practice the reference DNA amplification method. The DNA amplification method and the alternative plate method have equivalent sensitivity and either can be used. The non-PCR method is more tedious but can be used whenever a laboratory is not fitted out for PCR. The PCR and non-PCR approved regulatory methods both include a 5 h enrichment step after which selective inhibitors are added to suppress growth of non-target incurred background microflora.

The goal of this work was to demonstrate consistent detection of a single cell of *E. coli* O157:H7 in raw spinach. The results of these experiments are detailed herein.

## Materials and Methods

### Methods of Analysis

A disease outbreak isolate (*E. coli* serotype O157:H7, ATCC 43895), which produces both Shiga-like toxins I and II (Feng et al., 2001), was used as the target strain in this study. The food source was raw spinach obtained in two pound bags (West Creek, Richmond, VA, United States). The stock culture of *E. coli*, was grown 24 h to stationary phase at 37°C in Tryptic Soy Broth (Becton Dickinson and Company, DIFCO^TM^). This stock was diluted using sterile 1× PBS from 10^-1^ to 10^-8^. In preparing low level inoculum with a desired concentration of 1 cell/100 μL, the sample custodian first used the flow cytometer with *E. coli* O157 antibodies to determine the target cell concentration in a 10^-7^ dilution grown to stationary phase overnight. Triplicate measurements counted 18, 18, and 23 cells or an average of 19.7 ± 2.4 per 100 μL. A 1:19 dilution of this suspension was estimated to have an average concentration of 0.74 cells per 100 μL. TSA plates were inoculated with 100 μL of this stock suspension and incubated at 37°C for 24 h. The resulting colony counts from three plates (0,0,1 or 0.33 ± 0.47) were used for retrospective confirmation of the estimate but the 1:19 dilution was immediately used for inoculation of all positive samples, both reference BAM and the flow cytometry alternative.

Other aspects of the experimental process are detailed below including more specifics of sample preparation (see Experimental Design and Sample Preparation), the flow cytometer’s unusual features (see The Flow Cytometer and RAPID-B Reagents), an approved modification of the BAM reference method to assure fair comparison of sensitivity [see Bacteriological Analytical Manual Processing Method (Modified)], and an abbreviated protocol for sample processing before flow cytometric analysis (see Abbreviated Flow Cytometry Processing Protocol).

### Experimental Design and Sample Preparation

Recently we published successful completion of an FDA Level 2 validation study in raw spinach using the flow cytometry system and reagents specific for *E. coli* O157:H7 (Williams et al., 2015). The flow cytometer combined with the appropriately selective tagging reagent detects a few cells of *E. coli* O157:H7, but is insensitive to other *E. coli* serotypes and non-*E. coli* bacteria (Buzatu et al., 2015). Analysis takes 1 min with approximately two additional minutes required for an automated rinse procedure and reloading of the next sample. However, to achieve required sensitivity, eliminate flow cell occlusion, and reduce background interference from the spinach matrix required 18 sample preparation steps including photobleaching, centrifugation, filtration, and gradient centrifugation.

In a single case during the published Level 2 validation, when only a 5-h enrichment was specified, the rapid method failed to detect a single cell (Williams et al., 2015). To address this observation, the enrichment period was lengthened. Simultaneously many other details of the preparation method were simplified. Testing these modifications was another purpose of the work presented here. In addition, the design of an FDA Level 3 validation was used to assure that the method as modified could stand up to a rigorous comparison challenge and qualify for a multi-lab validation.

During further method optimization after the FDA Level 2 validation (Williams et al., 2015), we realized that large scale centrifugation, phloxine B photobleaching, and gradient centrifugation were not necessary to prevent confusion of spinach matrix particles with target cells that grow as rapidly as *E. coli*. In a preliminary experiment, growing the cells an hour and a half longer eliminated the need for preprocessing, particularly the concentration steps, and yielded results comparable to or better than the Level 2 experiments (preliminary data not shown—see Williams et al., 2015). Thus, this work used greatly abbreviated processing for the flow cytometry samples.

For this raw spinach test matrix, addition of a competitor strain was deemed unnecessary by the FDA expert advisor because up to 10^7^ non-pathogenic bacteria were already present per gram of spinach as normal flora (email sent to author from agency official on February 27, 2013^1^). The low inoculation level was intended to assess the detection limit for the novel flow cytometry method. Range-finding established its nominal failure at inoculations so low that the most likely explanation was failure to introduce even a single cell of the target pathogen.

Twenty-five samples of 25 g spinach each were processed and analyzed for each method (50 total). The two methods, the rapid and regulatory, were based on flow cytometer event counts and plate counts, respectively. For each method, five blank samples (i.e., containing no *E. coli* O157 cells) as well as 20 samples inoculated with target *E. coli* O157. The target cell inoculations were at a low number such that, for the experimental method, 25–75% fractional recovery (the percentage of nominally false negative results) would be obtained. In setting up the method comparison, 40 of the spinach samples were each inoculated with approximately one cell of *E. coli* O157:H7 per 100 μL as described in Section “Methods of Analysis.” Ten spinach samples representing negatives were each inoculated with 100 μL of sterile 1× PBS. Similarly, 40 spinach samples representing positives were each inoculated with 100 μL of the dilution from stock described in Section “Methods of Analysis.” In this study, the samples were refrigerator aged for 48 h, as described in Section “Sample Setup,” then incubated at 42°C in BHI broth for 6.5 h before analysis. We had determined from several years’ experimentation that these incubation conditions were optimal for injured *E. coli* cell recovery and early transition out of lag phase. These experimental designs, criteria, and procedures were based on requirements for an FDA Level 3 validation.

In this study, the reference method was adapted for a smaller 25 g sample as detailed in Section “Bacteriological Analytical Manual Processing Method (Modified).” This method modification was approved by Thomas Hammack, the FDA expert advisor in charge of new rapid method evaluation. Failure to modify the method and consequent addition of the larger volume of PBS appropriate for a 150–200 g sample led to catastrophic failure of the reference method because the one to two cells were much less likely to appear in the aliquot tested.

The rapid and alternative regulatory methods were being analyzed based on parallel samples rather than samples split after enrichment because the two used different enrichment media, different incubation temperatures, and different periods of non-selective enrichment. Therefore, comparison of sensitivity was only possible based on recoveries—that is, by comparing the number positive by the two methods for 20 nominally positive samples each. The required sensitivity was that the rapid alternative method should achieve equivalent or better recovery than the regulatory standard method.

### The Flow Cytometer and RAPID-B Reagents

The flow cytometer is a model A40 (Apogee, Hemel Hempstead, England, United Kingdom). Using an unusual flow cell design, the A40 achieves 130 nm optical resolution in low angle and high angle light scattering channels, performance particularly useful for detecting particles the size of bacteria (Wilkes et al., 2012). Its excitation source is a solid state 20 mW 488 nm (blue) laser. Fluorescence emission is detected at flow cytometry standard wavelengths: FL1 = 525 λ, FL2 = 575 λ, and FL3 = >610 λ. To maximize sensitivity for detecting small particle events, a photomultiplier tube is used for each light scatter and fluorescence emission channel. The electronic gains and voltages are factory calibrated so that, using a data acquisition protocol developed for the designated target (here, *E. coli* O157), the transmitted and excluded events are optimal and consistent. This enables sharing of method gate definitions among model A40 instruments (Wilkes et al., 2012).

In RAPID-B, specificity for detection of *E. coli* O157 is obtained by adding, 5 min before cytometric analysis, two reagents the composition of which is detailed in the Level 2 validation study (Williams et al., 2015). Briefly, Reagent A contains *E. coli* O157-specific purified polyclonal antibodies tagged with an FL1-emitting (green) fluorophore (Vivione Biosciences, LLC, Pine Bluff, AR, United States). Reagent B includes a mixture of components that prepare the bacterial cell surfaces freeing epitopes for easy access by the antibody and a membrane-impermeable DNA-intercalating dye then emits in the Fl3 (red) channel. When a bacterium dies, its cell membrane becomes porous so that the dye penetrates in to the DNA and the cell glows red, a signal that it is no longer viable even if it is tagged with the target-specific antibody. Enumeration of target cells is usually counted as events that scatter the incident blue light in expected intensities and that glow green but not red.

### Bacteriological Analytical Manual Processing Method (Modified)

As stated above, parallel samples were prepared for BAM 4a analysis using the same inoculum and procedures as for the flow cytometry procedure. The samples were then processed, using a modification of the standard regulatory procedure. Since we only used a 25 g sample of spinach per bag, instead of a 200 g sample amount (typically specified for composite samples), a proportionally lower amount of sterile PBS was added (i.e., 25 mL) to each sample before they were placed on a shaker-incubator for 5 min. 20 mL of 2× modified buffered peptone water pyruvate (mBPWP, Remel, Labsource, Romeoville, IL, United States) was added to each before placing them back into the incubator at 37°C for 5 h. At this point the BAM4a specifies addition of inhibitors to which background microflora are typically more sensitive than the target *E. coli* O157:H7. We added 333 μL of an ACV cocktail (Acriflavine, Cefsulodin, Vancomycin) containing Acriflavine and Cefsulodin at 7.5 × 10^-4^ g/mL each and Vancomycin at 6.0 × 10^-4^ g/mL, all from MP Biomedicals, LLC, Solon, OH, United States. The volume added was adjusted proportionally for the smaller 45 mL suspension volume for single 25 g spinach samples. All subsequent processing for the regulatory samples was the same as outlined in the BAM manual (Feng et al., 2011).

### Abbreviated Flow Cytometry Processing Protocol

As mentioned above, this work used greatly abbreviated processing for the flow cytometry samples, which simplified the sample handling protocol. Compared to 18 previously used, five sample setup, pre-analysis, and analysis steps are required in this abbreviated method to determine *E. coli* O157:H7. (Only the first of the six steps listed below under Setup would be used when analyzing real-world unknowns for incurred contamination.) The TTR for 25 samples was equal to that in the Level 2 work (Williams et al., 2015), even with the extra 1.5 h enrichment, because of the smaller number of preparation steps.

#### Sample Setup

1. Twenty-five grams of spinach were weighed into each of a specified number of individual sterile Whirl-Pak filter bags.

2. A 100 μL volume of either *E. coli* O157:H7 ATCC 43895 or sterile 1× PBS was inoculated into each sample bag.

3. The inoculum was massaged into the spinach leaves and placed in a refrigerator set at 3–5°C for 48 h.

4. To confirm cell counts, 100 μL of the stock dilution used to inoculate each bag of spinach, was plated onto triplicate TSA plates and incubated at 37°C overnight (17 h).

5. Forty-eight hours later a 75 mL aliquot of sterile, preheated BHI (stored at 42°C overnight) broth was added to each sample to be analyzed on the flow cytometer.

6. Each sample to be analyzed using flow cytometry was then massaged by hand, 10× each, before being placed in the 42°C incubator for 6.5 h to allow for growth of the bacteria.

#### Pre-analysis

7. After the 6.5 h enrichment, each sample was massaged by hand one final time to ensure homogeneous suspension of the bacteria.

8. A 1 mL aliquot from each suspension was filtered into a sterile 2 mL microcentrifuge tube using a 5-μm pore size 25 mm diameter PVDF syringe filter (Millipore Corporation, Billerica, MA, United States; Becton Dickinson and Company, Sparks, MD, United States).

9. A 100-μL aliquot of the filtrate was mixed with 650 μL of sterile 1× PBS, 240 μL of Reagent B, and 10 μL of Reagent A. This was gently vortexed at a setting of 2 on a Vortex-Genie 2 fitted with a 48 hole foam rack (Daigger, Wheaton, IL, United States) at lab ambient temperature for 5 min before analysis.

#### Analysis

10. Samples were analyzed every 3 min on the flow cytometer (1 h and 15 min for 25 samples). Details of instrumental setup, operation, and cleanup between samples can be found at Williams et al. (2015, section 2.4). No events appeared in the final counting gate for non-inoculated samples.

## Results

In these studies, neither method reported false positives. The flow cytometry method TTR for 25 samples was 9 h, including the 6.5 h incubation time; if a sample reported an ambiguous result it was possible to repeat analysis the next day. A result was considered ambiguous if the counted number of events greatly exceeded typical negatives (0 or 1) and was far fewer than typical positives (20s, hundreds, thousands). For the BAM, TTR was much longer, 51–60 h. The plate-based and PCR regulatory method reported a much higher percentage of negative results for nominally positive samples, 16 of 20 (80%), compared to the rapid method’s 7 of 20 (35%). An explanation of results in **Table 1** is included in the paragraphs that follow.

**Table 1:** Results using the In-House Level 3 Validation experimental design.

Sample Number	Cytometry Cell Counts (Cts.; Re-Cts.)	Cytometry I.D.^∗^	BAM I.D.^∗^	“Positive” Samples	Negative Samples
1	0	–	–		–
6	0	–	–		–
9	0	–	–		–
13	1	–	–		–
21	0	–	–		–

2	13;13↑	+	+	+	
3	0	–	–	+	
4	0	–	–	+	
5	86	+	–	+	
7	0	–	–	+	
8	0	–	–	+	
10	40	+	–	+	
11	157	+	+	+	
12	6;31↑	+	–	+	
14	0	–	–	+	
15	1	–	–	+	
16	12220	+	–	+	
17	3201	+	–	+	
18	312	+	–	+	
19	0	–	+	+	
20	413	+	+	+	
22	1713	+	–	+	
23	520	+	–	+	
24	1591	+	–	+	
25	2780	+	–	+	

*^∗^Bold red “–” symbols indicate inconsistency when compared to “Positive” Sample I.D.**^↑^**For the rapid method, the second number in a pair represents the event count from a reprocessed aliquot of sample that was left at ambient laboratory temperature overnight. Reprocessing was done in the two cases when initial screens were ambiguous. Counts were as 476 measured after the 6.5 h enrichment. Re-counts were measured after refrigeration overnight.*

The Sample Numbers for Cytometry and BAM analysis samples correspond with respect to the nominal “Positive” Sample I.D., but individual samples are not the same for BAM and Cytometry nor do they necessarily correspond for positive samples with respect to actual I.D. because an inoculated, nominally positive sample might or might not have actually contained any *E. coli* O157 cells. Lack of correspondence between nominally positive Cytometry and BAM is possible because they were parallel, not split after enrichment. Experimental design causing this potential discrepancy was necessary because, unlike the BAM, the Cytometry enrichment conditions were fully optimized for consistent recovery of very low level contaminations measurable within the same day of sample arrival and without strain isolation.

**FIGURE 1:**
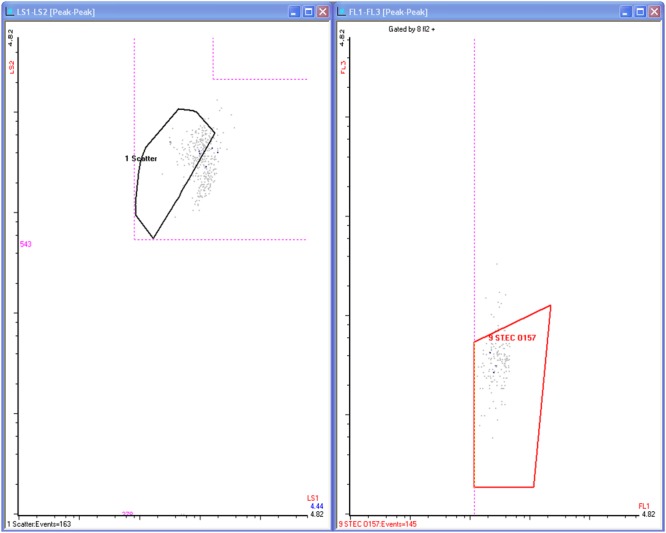
This shows two of the flow cytometer screens, dot plots for light scatter and fluorescence emission for a typical positive sample in this work. The cluster of dots appearing within the FL1 (green) vs FL3 (red) fluorescence gate are events transmitted through multiple gates and each dot represents detection of a viable *E. coli* O157 cell. They number 145, seen at the left bottom of the plot.

Initial flow cytometry results were ambiguous in two cases, Sample 2 and Sample 12. These samples were reanalyzed the next day after overnight refrigeration. Flow cytometer Samples 2 and 12 were ambiguous because, respectively, the 13 or 6 counted events were so few in comparison to other positive samples, but the observed events appeared to cluster in the middle of the final counting gate like real target cells (See **Figure 1**). After reprocessing, they were confirmed as true positives. Because of the characteristic location of the signals and the absence of nearby matrix signals, these 13 or 31 counts, respectively, were no longer ambiguous.

A substantial portion of the BAM TTR involved overnight plate confirmation of presumed positives by re-culturing isolates on Tellurite Cefixime Sorbitol MacConkey Agar plates (TC-SMAC). The regulatory method did not report questionable results in these tests so BAM repeat analyses were not warranted.

## Discussion

For these experiments, the cytometry-estimated and plate-confirmed inoculation levels were 0.74 ± 0.43 and 0.25 ± 0.43 cfu/100 μL, respectively. That is, the majority of the nominally positive samples were actually inoculated with only one or zero viable *E. coli* O157:H7 cells. Two viable cells, appearing more than two standard deviations above the average, would occur in approximately 5% of the cases, 1 of 20 samples. The flow cytometry method achieved positive results in 65% of the nominally positive inoculations, which met an FDA method validation criterion that inoculation levels be chosen such that the rapid method achieves 25–75% positive recovery. The 65% observed recovery by the experimental rapid method is consistent with an explanation that it failed only in samples where zero recoverable cells were inoculated. The BAM 4a regulatory method yielded 4/20 (20%) positive samples. These results represent a statistically significant performance difference between the flow cytometry method and the reference method (*p* = 0.01 in a two-sided Fisher’s exact test). That is, the flow cytometry method was more sensitive than the regulatory method in these experiments is confirmed with statistically significant certainty.

The lack of ambiguity implied that reduced BAM sensitivity might be explained by factors influencing recovery that preceded selective plate confirmation steps (i.e., no observable colonies to recover for more specific plating). A potential explanation for BAM 4a reduced sensitivity is that its enrichment period prior to introduction of growth inhibitors that depress competitive microflora may be too short to allow consistent recovery of a single, stressed target cell. The BAM PCR-based and plate-based regulatory methods both add selective growth inhibitors at five h post-inoculation. The lag phase is known to lengthen when bacteria are isolated and/or stressed (Feng et al., 2011). These results suggest the detection limit for the current BAM 4a official method may be greater than one cell and that the flow cytometry method with a 6.5 h non-competitive enrichment may be more sensitive. BAM 4a might become as reliably sensitive as the flow cytometry method by postponing addition of growth inhibitors an extra hour and a half, a strategy that assumes such postponement would not lead to interference at a later stage of the assay from the increased abundance of competitive microflora. The flow cytometer method appears to be less vulnerable to the effects of competitive microflora, perhaps because its analysis is completed after 6.5–9 h, responses are measured for each cell, and unlike the BAM PCR method there is no minimum number of cells (required by PCR prior to DNA amplification) so that signal is sufficient to record a positive.

For any sample that the flow cytometer determined to be positive, there were at least six counts although typically there were many more (1920 ± 3280). Each sample defined as blank by the key showed counts of only 0 or 1. Based on the sample key, such samples were correctly classified as negatives. There were no confirmed false positives by either method. Of seven nominally positive samples deemed negative by the flow cytometry method, six reported zero counts and one reported a single count. The nominal positive/actual negatives and true negatives averaged 0.16 ± 0.37 counts. The minimum signal-to-noise ratio for the smallest count eventually deemed positive was 6/0.16 = 38. The average signal-to-noise ratio was 1920/0.16 = 12,000.

The four orders of magnitude range of cells counted after the 6.5 h enrichment were estimated to have arisen in 95% of the cases from a single viable cell inoculated. The fact that up to 2.5 orders of magnitude difference can result from apparently similar circumstances underscores the critical contribution of cell stress in determining how quickly it is feasible to enrich the cell numbers during such experiments. Evidence that bacterial cell isolation is a major contributor to stress (and thus that a lengthened lag phase might occur) can be inferred by comparison to results from our other publications in this series (Buzatu et al., 2013; Williams et al., 2015). In those experiments when inoculation levels were only a little higher (14 cells and two to four cells, respectively) it was possible to observe clusters of counts estimated to arise from low single digit inoculations. That is, when a particular number of cells was inoculated, the resulting cell count after short-term enrichment was fairly consistent among samples and the different clusters of similar cell counts appeared to be a linear function of a hypothesized low-single-digit number inoculated. In other words, when a cell was not completely alone, its lag phase duration and consequent post-enrichment cell counts were more predictable. The hypothesized solitary cell phenomenon causing a lengthened lag phase is consistent with the concept of “quorum sensing” amongst bacterial cells (Miller and Bassler, 2001).

## Conclusion

The flow cytometry method for determining *E. coli* O157:H7 contamination in raw spinach provided results in 9 h for 25 samples compared to 60 h required by the reference regulatory method. Comparison of results for nominally positive samples showed that the flow cytometry method is significantly more sensitive for detecting *E. coli* O157:H7 in raw spinach than the BAM method. Using only four steps of sample preparation and analysis, the cytometry method detected single cell contamination with a more than 38× signal-to-noise ratio. In summary, compared to BAM 4a, the flow cytometry method (1) takes much less time, (2) is much less labor intensive, (3) is as accurate, and (4) is more sensitive. For these reasons, the abbreviated cytometry protocol with flow cytometry detection has potential utility as a screening tool to detect *E. coli* O157:H7 in foods.

## Author Contributions

DB and JW are principal investigators of the project. AW and WC are co-PI’s who performed the experiments. AW and JW wrote the manuscript, with assistance from DB in editing. SR set up inoculations, validated the inoculation levels by plate count, and served as sample custodian. PA assisted in the experiments and helped with the editing.

## Disclaimer

The views presented do not necessarily reflect opinions or official policy of the U.S. Food and Drug Administration.

## Conflict of Interest Statement

Some of the authors are inventors of technologies, which have been patented by the US FDA, related to this paper, and for which royalties can be received. The other authors declare that the research was conducted in the absence of any commercial or financial relationships that could be construed as a potential conflict of interest.

## Footnotes

1 Thomas Hammack, email message sent to authors, February 27, 2013.
